# Tele-Mentored Handheld Ultrasound System for General Practitioners: A Prospective, Descriptive Study in Remote and Rural Communities

**DOI:** 10.3390/diagnostics13182932

**Published:** 2023-09-13

**Authors:** Yu-Jing Zhou, Le-Hang Guo, Xiao-Wan Bo, Li-Ping Sun, Yi-Feng Zhang, Hui-Hui Chai, Rui-Zhong Ye, Cheng-Zhong Peng, Chuan Qin, Hui-Xiong Xu

**Affiliations:** 1 Department of Medical Ultrasound, Jinshan Hospital, Fudan University, Shanghai 201508, China; zhouyujing006@126.com; 2 Department of Medical Ultrasound, Shanghai Tenth People’s Hospital, Tongji University, Shanghai 200072, China; gopp1314@hotmail.com (L.-H.G.); boxiaowan0908@126.com (X.-W.B.); sunliping_s@126.com (L.-P.S.); zhangyifeng@tongji.edu.cn (Y.-F.Z.); 18895687926@163.com (H.-H.C.); 3 Shanghai Engineering Research Center of Ultrasound Diagnosis and Treatment, Shanghai 200072, China; 4 Department of Ultrasound Medicine, Zhejiang Provincial People’s Hospital (Affiliated People’s Hospital, Hangzhou Medical College), Hangzhou 310014, China; yee981120@139.com; 5 Department of Ultrasound, Karamay Central Hospital, Karamay 834000, China; 6 Department of Ultrasound, Zhongshan Hospital, Fudan University, Shanghai 200032, China; xuhuixiong@126.com

**Keywords:** tele-mentored, handheld ultrasound, general practitioners, rural and remote

## Abstract

Background: Rural general practitioners (GPs) have insufficient diagnostic information to deal with complex clinical scenarios due to the inequality in medical imaging resources in rural and remote communities. The objective of this study is to explore the value of a tele-mentored handheld ultrasound (tele-HHUS) system, allowing GPs to provide ultrasound (US) services in rural and remote communities. Methods: Overall, 708 patients underwent tele-HHUS examination between March and October 2021 and March and April 2022 across thirteen primary hospitals and two tertiary-care general hospitals. All US examinations were guided and supervised remotely in real time by US experts more than 300 km away using the tele-HHUS system. The following details were recorded: location of tele-HHUS scanning, primary complaints, clinical diagnosis, and US findings. The recommendations (referral or follow-up) based on clinical experience alone were compared with those based on clinical experience with tele-HHUS information. Results: Tele-HHUS examinations were performed both in hospital settings (90.6%, 642/708) and out of hospital settings (9.4%, 66/708). Leaving aside routine physical examinations, flank pain (14.2%, 91/642) was the most common complaint in inpatients, while chest distress (12.1%, 8/66) and flank discomfort (12.1%, 8/66) were the most common complaints in out-of-hospital settings. Additionally, the referral rate increased from 5.9% to 8.3% (kappa = 0.202; *p* = 0.000). Conclusions: The tele-HHUS system can help rural GPs perform HHUS successfully in remote and rural communities. This novel mobile telemedicine model is valuable in resource-limited areas.

## 1. Introduction

According to the World Health Organization (WHO), simple ultrasound (US) or X-rays can cover the needs of approximately two-thirds of diagnostic imaging services [1]. However, there is a widespread inequality in global medical resources, with US imaging in remote areas being resource-intensive, which makes high-quality US services inaccessible for the residents of such areas [2]. Instead, they are required to travel to distant general medical centers for US services. Additionally, the ageing of the global population is increasingly becoming a serious issue; it is becoming more difficult for numerous elderly people in remote rural areas to receive timely medical services.

With the development of newer technologies, US equipment has become miniaturized [3,4]. Novel handheld ultrasound (HHUS) devices are the latest type of US equipment. Due to its portability, low cost, and good reproducibility, HHUS has become recognized for its utility in resource-limited settings, such as pre-hospital emergency environments, inpatient and outpatient settings, and emergency departments [2,3,5,6,7]. HHUS is expected to provide a sufficient level of US services in remote areas.

However, the application of HHUS poses serious challenges. Although rural doctors may receive basic US training with HHUS devices, they usually cannot perform HHUS examinations skillfully at the outset. Additionally, insufficient operating backup support from US experts may discourage them from performing HHUS.

With the development of telemedicine technology, studies have demonstrated that novices without US experience can successfully perform US examinations with the guidance of remote experts [8,9,10,11]. It should be noted that most studies on tele-mentored ultrasound (tele-US) were not based on HHUS.

In recent years, with the advancement of US technology, the makers of US devices have developed portable handheld equipment connected to smart mobile devices that allow real-time US images to be shared remotely through commercial chat software or digital software platforms [4,12]. However, few studies have been conducted using this tele-mentored handheld ultrasound (tele-HHUS) system. A pilot study demonstrated that the use of a tele-HHUS system by healthcare providers under the supervision of obstetricians improved access to antenatal ultrasound for women in rural Ethiopia [13].

In this instance, the healthcare providers were exclusively focused on a single clinical scenario. However, rural general practitioners (GPs) usually encounter complex clinical scenarios. Whether tele-US could help rural GPs perform HHUS successfully remained unconfirmed. Therefore, we conducted this multicenter study in real-world conditions to explore the value of a tele-HHUS system in rural and remote communities.

## 2. Materials and Methods

### 2.1. Design

**Figure 1:**
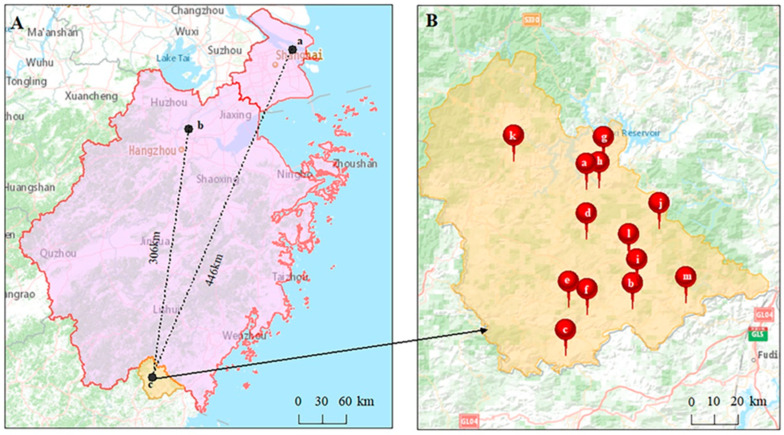
Picture (**A**) shows that the straight-line distances between Taishun county and Shanghai Tenth People’s Hospital and Zhejiang Provincial People Hospital are 446 km and 306 km, respectively. (a) Shanghai Tenth People’s Hospital; (b) Zhejiang Provincial People’s Hospital; (**c**) Taishun County. Picture (**B**) shows the geographical distribution of the 13 community hospitals in Taishun County. (a) Siqian Town Central Health Center; (b) Shiyang Branch of Taishun County Hospital of Chinese Medicine; (c) Sankui Branch of Taishun County People’s Hospital; (d) Sixi Branch of Taishun County People’s Hospital; (e) Xiaocun Branch of Taishun County People’s Hospital; (f) Fengyang Branch of Taishun County People’s Hospital; (g) Baoyang Township Health Center; (h) Zhuozhai Village Clinic; (i) Nanpuxi Town Health Center; (j) Nanpuxi Town Lianyun Health Center; (k) Pengxi Town Central Health Center; (l) Sixi Town Hengkeng Health Center; and (m) Xiyang Town Health Center.

This prospective, descriptive study was conducted across thirteen primary hospitals in Taishun County and two tertiary-care general hospitals. These community hospitals were 446 and 306 km away from the tertiary-care hospitals, respectively (Figure 1). This study was approved by the ethics committee of the Shanghai Tenth People’s Hospital (approval number: SHSY-IEC-4.1/20–65/01).

### 2.2. Participants

The study comprised 28 rural doctors from 13 primary hospitals, who were all GPs without any US experience. They had 5–15 years of clinical work experience, usually diagnosed and treated frequent and common diseases, recorded data of chronic diseases, and followed up regularly. The US diagnoses were supported by 9 independent remote experts with at least 5 years of experience in US using the tele-HHUS system (STOCK, Chengdu, China). All the participants were listed in Appendix A.

### 2.3. Patients

Between March and October 2021 and March and April 2022, 726 patients underwent tele-HHUS examination. The inclusion criteria were as follows: (a) age ≥ 18 years; (b) various indications for US examinations, such as establishing a disease diagnosis, follow-ups, and routine physical examinations. The exclusion criterion was incomplete information.

### 2.4. Training Protocol

The rural GPs received face-to-face lessons from experienced US specialists in Taishun County. The training content was carefully designed according to the clinical scenarios faced by local GPs. The training course included 3 days of theoretical learning and hands-on practice with HHUS and 2 weeks of US internship at the local second-level hospital. The course syllabus included the principles of ultrasonography and instrument operation, abdominal and cardiovascular US anatomy, US assessment of abdominal trauma and acute abdomen, and US assessment of hypotension or hypoxemia. The rural GPs who finally passed the theory and practical exams received a certificate of qualification, which was approved by the local health commission.

### 2.5. Tele-HHUS System

**Figure 2:**
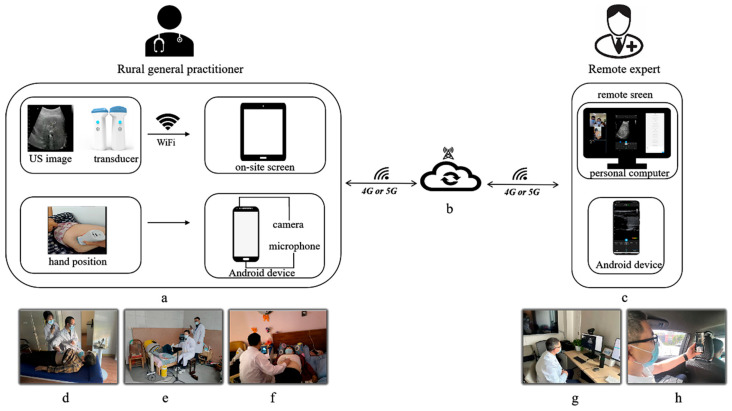
Tele-HHUS system: (**a**) set-up for the rural general practitioner; (**b**) cloud platform; (**c**) set-up for the remote expert; (**d**) outpatient clinic; (**e**) patient’s home; (**f**) nursing home; (**g**) remote screen on a personal computer in the expert’s office; (**h**) remote screen on an Android smartphone in the expert’s car.

The equipment used in this study was a HHUS system (STOCK, Chengdu, China). This system consisted of a wireless low-frequency (2–5 MHz) HHUS transducer (this transducer is also used for 2D echocardiography); an “on-site screen” (a proprietary tablet) and a “camera and microphone” (an Android device) for the use of the rural GPs; and a “remote screen” (a personal computer or Android device, such as a tablet or smartphone) for the experts, along with the network that connected them (Figure 2).

The US transducer was connected with the “on-site screen” through the transducer’s built-in Wi-Fi. A custom mobile application or web-based system was integrated with the workstation, which had a doctor-side and a patient-side and could carry out all the telemedicine work. Real-time US images were displayed on the screen and uploaded to the cloud. The “camera and microphone” utilized an additional on-site Android device, such as a smartphone, which allowed for simultaneous visualization of the operator’s hand position and voice interaction with remote experts.

The real-time US images, hand position of the operator, and voice interactions were uploaded to the cloud and observed by the remote experts using the “remote screen”. Data transmission was based on a 4G or 5G network (with a downlink/uplink rate of 300 Mbps).

### 2.6. Scanning Procedure

A GP performed HHUS scans of each patient with online guidance from a remote expert. Remote experts could guide the GPs in utilizing the correct position, acquiring standard images, improving image quality, and deriving potential diagnoses. Additionally, they could communicate with the patients, together with the GP. When either of them detected a positive finding, both could save the image for further analysis. Finally, an expert completed the US report and uploaded it to the cloud. Subsequently, the rural GP could download and print it.

### 2.7. Measurements and Outcomes

For each patient, we recorded the location of the tele-HHUS scanning, primary complaints, the clinical diagnosis, and US findings. Recommendations based on clinical experience alone, and those based on clinical experience with additional tele-HHUS information, were compared. The recommendations were categorized as follows: (1) follow-up; (2) referral for other medical interventions (e.g., additional imaging examination or laboratory testing, or further therapy, as appropriate).

### 2.8. Statistical Analyses

Statistical analyses were performed using SPSS v26.0 (IBM Corp., Armonk, NY, USA) and Microsoft Excel 2019 (Microsoft Corp., Redmond, WA, USA). Quantitative variables are presented as mean ± standard deviation and range. Categorical variables are presented as numbers and percentages.

## 3. Results

### 3.1. Patients and Locations of Tele-HHUS

A total of 708 patients were included in this study (mean age, 60.7 ± 14.6 years). Of these, 43.7% (310/708) were males and 56.7% (398/708) were females. Eighteen patients were excluded due to incomplete information. Tele-HHUS examinations were performed in in-hospital settings (outpatient clinics: 90.6%, 642/708) and out-of-hospital settings (9.4%, 66/708). The out-of-hospital settings included patients’ homes (4.8%, 34/708), committee centers (2.4%, 17/708), nursing homes (1.6%, 12/708), and mobile service vehicles (0.4%, 3/708).

Leaving aside routine physical examinations, flank pain (14.2%, 91/642) was the most common reason for patients to seek treatment in in-hospital settings, while chest distress (12.1%, 8/66) and flank discomfort (12.1%, 8/66) were the most common complaints in patients in out-of-hospital settings (Table 1).

Among 34 patients who received tele-HHUS at home (whose average age was 78.4), 2 were blind with glaucoma, 1 had a hand disability, 4 suffered from lower-limb paralysis, 6 had hypertension, 6 had a history of stroke, and 6 suffered from diabetes. A total of 64.7% (22/34) of these patients had not undergone US examination for three to five years.

### 3.2. US Examinations and Results

**Figure 3:**
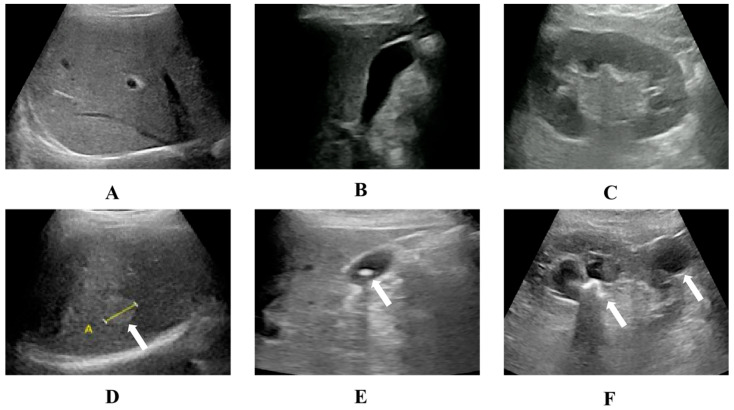
Illustrative examples of ultrasound findings using the tele-HHUS system. Normal liver, gallbladder, and kidney (**A**–**C**). Hepatic hemangioma (arrows) was identified in a 51-year-old female who presented to an outpatient clinic after suffering from abdominal discomfort for 3 days. She had a history of hepatic hemangioma (**D**). Gallstones (arrows) were identified in a 68-year-old man at his home after he complained of general discomfort (**E**). A kidney stone with hydronephrosis and renal cyst (arrows) was identified in a 76-year-old man at his home after he complained of flank discomfort (**F**).

A total of 16 anatomical sites were scanned, and more than 30 diseases were diagnosed using the tele-HHUS system (Table 2 and Table 3). The kidneys (38.5%, 378/987) and liver (35.7%, 352/987) were the organs most commonly scanned. Fatty liver (23.0%, 173/752) and kidney stones (20.2%, 152/752) were the most common abnormalities identified. Representative normal and anomalous US images are depicted in Figure 3.

### 3.3. Changes in the Treatment Plans

With the aid of the tele-HHUS system, 59 (8.3%) patients were recommended referrals based on US findings. By contrast, when diagnoses were based on clinical experience alone, only 42 (5.9%) patients were referred. Of these, 13 patients were recommended referrals with the aid of the tele-HHUS system. Furthermore, tele-HHUS helped modify the decision to consider follow-ups based on clinical experience alone in another 46 patients (Table 4).

## 4. Discussion

In this prospective, descriptive study, we showed a wide application for tele-HHUS both in in-hospital settings and out of hospital in remote communities. Using the tele-HHUS system, remote US experts could help rural GPs perform US successfully in different real-world conditions. Hundreds of local villagers from 13 communities received a high-quality ultrasound diagnosis service and definite referral recommendations in resource-limited settings. We demonstrated that the tele-HHUS system, supported by a network connection to remote experts, is valuable not only for the GPs but also for the local patients and the entire healthcare environment in remote and rural communities.

In particular, we proved the benefits of the tele-HHUS system for patients in out-of-hospital settings. For example, seeking high-quality healthcare is more difficult for elderly people with reduced mobility (including disability and lower limb paralysis caused by stroke) and multiple comorbidities. Long distances, high costs, and lack of good medical resources nearby hindered their access to timely medical check-ups. Although rural GPs may visit and provide basic medical care, US examinations are extremely difficult to perform in such settings. Therefore, based on our data, 75% of the patients had not undergone US examination for more than 5 years. With the aid of the tele-HHUS system, elderly people can undergo point-of-care US performed by rural GPs in their homes instead of visiting hospitals. Visualization of their internal physical condition, especially for patients with symptoms, could be used to determine whether they require referrals to higher centers. With timely visual information, the diagnostic efficiency for elderly patients is improved, and the diagnosis time of potential diseases is significantly advanced. One study showed that a handheld ultrasound device could effectively improve clinical decision-making at the point of care of geriatric patients [14], which makes us more confident using the tele-HHUS system in the homes of the elderly.

In the present study, we described wide potential applications for the tele-HHUS system to access multiple organs. We believe that most patients could benefit from this novel system. For example, a 37-year-old man with severe flank pain visited his nearest clinic; however, there was no US device equipped with the tele-HHUS system at that clinic. Using the tele-HHUS system, the rural GP confirmed the presence of kidney stones with hydronephrosis, and the patient was immediately treated appropriately. Similarly, a 67-year-old man with severe abdominal pain and abdominal distention was immediately diagnosed with acute cholecystitis using the tele-HHUS system and received appropriate treatment. Therefore, owing to the universal applicability of the tele-HHUS system, we believe that it could play a key role in providing basic healthcare services for the public, especially in resource-limited settings [15]. Many studies have shown that as a new portable diagnostic tool, HHUS has the same diagnostic efficiency as conventional ultrasound in the diagnosis of diseases of the heart, liver, lungs, and other organs [5,16,17,18]. HHUS has unlimited diagnostic potential, especially in acute and critically ill patients [19,20]. Therefore, strong evidence is provided for the widespread efficacy of the tele-HHUS system.

Based on our results, we believe that rural GPs can benefit from this novel telemedicine model. Using a network connection, the online guidance of experts is a good method for acquiring professional knowledge, one that is conducive to improving the US skills of rural GPs. At the same time, it mitigates the shortage of medical resources in rural primary hospitals. For example, some clinics equipped with US devices only provide US services periodically (e.g., once a week or less frequently) because of the unavailability of full-time sonographers. Furthermore, some clinics do not have US devices. Therefore, the government provides standalone HHUS devices (without networking) and relatively brief training for rural GPs in the hopes that they can fill the gap in US services. However, the GPs may not be proficient at performing US. We believe that rural GPs require continuous instruction before being able to perform US with the requisite skill. Therefore, the tele-HHUS system using remote experts can not only increase confidence in clinical diagnoses, but also continuously train rural GPs to perform better US examinations in order to mitigate the shortage of sonographers in remote communities.

With the advancement of technology and the development of health devices, mobile health (mhealth) has become an emerging field [21]. The significance of mhealth is that monitoring and intervention can be achieved whenever and wherever acute and chronic diseases occur [22]. In medical imaging, the miniaturization of US equipment and imaging on smart phones is at the forefront of mhealth. In our study, the tele-HHUS system is fully utilized to illustrate the effective synergy between mhealth and US, especially in specific scenarios such as homes and nursing homes, effectively improving vulnerable groups’ healthcare. A previous study indicated that mhealth and portable obstetric US interventions could help reduce maternal and neonatal mortality and morbidity in low- and middle-income countries [12], and also suggested that the development of mhealth in developing countries or remote areas is on the rise. Based on our research, a telemedicine model using the tele-HHUS system could form a model for promoting mhealth in remote areas. In resource-limited settings, mhealth diagnoses are strongly associated with patient referral outcomes and may be associated with improved outcomes in treatment. In the telemedicine model, the demand for healthcare in the medical industry is alleviated, and the level of healthcare in remote areas is improved.

Compared with other similar studies, our tele-HHUS model displayed some advantages. First, the system was synchronously supported using a 4G or 5G network so that remote experts could support rural GPs in a timely manner. By contrast, in two previous studies on HHUS-based remote screening for heart disease in the community, experts interpreted images asynchronously on a web-based system within a 2-day period, which significantly delayed the diagnoses [23,24]. Second, US scanning is both real time and dynamic. Therefore, 4G or 5G networks with extremely low network latency can provide a smooth experience for both the experts and rural GPs. Third, synchronous US video signals were uploaded directly from the HHUS device. However, in other studies, US images were indirectly acquired for remote experts by capturing the US device screen using commercially available video chat software [2,11]. Undoubtedly, the original videos provide a higher image quality. Fourth, the HHUS device is small and lightweight, which makes a dedicated screen unnecessary. The US image can be displayed on a handheld tablet or smartphone. Therefore, the proposed model can be more conveniently deployed. By contrast, in a previous study, robot-assisted US diagnostic systems used for tele-US were bulky, heavy, and complex [25,26,27,28]. A comparison of the tele-HHUS system with other existing systems is shown in Table 5.

The present study has some limitations. First, patients’ and rural doctors’ satisfaction with the tele-HHUS system was not evaluated. In future studies, questionnaires can be designed to fill this gap in knowledge. Second, there was no follow-up information concerning the patients, which makes it difficult to ascertain whether the changes in clinical management based on the tele-HHUS system were effective. Telephonic follow-up may be a feasible approach in future studies. Third, the tele-HHUS system is currently free of charge. However, it is necessary to discuss if it should be charged for, as well as investigating its cost performance. Fourth, the training protocol in this study was based on the experiences of individual experts. However, there is no consensus on a standard protocol for HHUS training [29]. A uniform and effective HHUS training program is essential before it can be recommended for broader applications. Fifth, the sample size of patients with chest distress was small. However, we wanted to perform focused cardiac ultrasound for these patients so as to provide more imaging information for clinical diagnosis, even though echocardiography is complicated, requiring a special investigation methodology. Therefore, this study can only be considered an exploration; further relevant large-scale, high-quality research is needed in the future. Finally, there was no control group, because this was a descriptive study. In further studies in the future, we will set up control groups for specificity and sensitivity analyses.

## 5. Conclusions

In conclusion, the tele-HHUS system is valuable in remote and rural communities. With the advances in mobile technology and digitization of healthcare, HHUS combined with 4G or 5G networks connected rural GPs and remote experts effectively to improve access to ultrasound services in rural communities. This novel mobile telemedicine model is worth popularizing and utilizing in resource-limited areas.

## Figures and Tables

**Table 1:** Patients’ basic information.

Setting	Number of Patients (%)	Mean Age, Years (Range) ^a^	Patients’ Gender Distribution (%)	Main Complaints (%)
In-hospital	642 (90.6%)	59 ± 14.1 (21–93)	Male 273 (42.5%) Female 369(57.4%)	Physical examination 164 (25.5%) Flank pain 91(14.2%)
Out-of-hospital	66 (9.4%)	75 ± 10 (51–101)	Male 37 (56%) Female 29 (43.9%)	Physical examination 29 (43.9%) Chest distress 8 (12.1%) Flank discomfort 8 (12.1%)

^a^ The data are expressed as means ± standard deviation, with ranges in parentheses.

**Table 2:** Tele-HHUS scans performed for various systems and specific organs.

	Scans Performed for Specific Organs	Number of Tele-HHUS Scans (%)
Digestive system	Liver	352 (35.7%)
	Gallbladder	78 (7.9%)
	Spleen	49 (5.0%)
	Pancreas	45 (4.5%)
Urinary system	Kidney	378 (38.5%)
	Ureter	17 (1.7%)
	Prostate	16 (1.6%)
	Bladder	8 (0.8%)
	Adrenal gland	2 (0.2%)
Gynecology and obstetrics	Uterus	27 (2.7%)
	Ovary	5 (0.5%)
	Pelvic cavity	4 (0.4%)
Circulatory system	Heart	4 (0.4%)
Other	Pericardial cavity Abdominal cavity Thoracic cavity	1 (0.1%) 1 (0.1%) 1 (0.1%)
	Total	987 (100.0%)

**Table 3:** Summary of tele-HHUS findings.

Tele-HHUS Findings	Number (%)
Fatty liver	173 (23.0%)
Kidney stones	152 (20.2%)
Renal cyst	112 (14.9%)
Renal crystallization	66 (8.8%)
Hepatic cyst	57 (7.6%)
Gallstone	37 (4.9%)
Hydronephrosis	30 (4.0%)
Hepatic hemangioma	18 (2.4%)
Hepatolith	17 (2.3%)
Gallbladder polyps	13 (1.7%)
Intrahepatic calcification	12 (1.6%)
Liver lesion	8 (1.1%)
Hysteromyoma	8 (1.1%)
Benign prostatic Hyperplasia	7 (0.9%)
Nephrarctia	5 (0.7%)
Liver solid mass	4 (0.5%)
Ovarian cyst	4 (0.5%)
Pericardial effusion	4 (0.5%)
Ureteral calculus	3 (0.4%)
Hydroureter	3 (0.4%)
Splenomegaly	2 (0.3%)
Dilated intrahepatic bile ducts	2 (0.3%)
Pregnancy	2 (0.3%)
IUD location	2 (0.3%)
Predominant left ventricular systolic dysfunction	2 (0.3%)
Adrenal occupying lesion	2 (0.3%)
Other ^a^	7 (0.7%)
Total	752 (100%)

^a^ Other: in this category rare findings were listed, e.g., acute cholecystitis, renal mass, bladder stone, pelvic mass, adenomyosis, pleural effusion, and ascites. Tele-HHUS: tele-mentored handheld ultrasound.

**Table 4:** Referrals based on clinical experience alone and referrals based on tele-HHUS findings.

	Based on Tele-HHUS Findings		
Based on Clinical Experience Alone	No Referral	Referral	Total
No referral	620 (87.5%)	46 (6.5%)	666 (94.0%)
Referral	29 (4.1%)	13 (1.8%)	42 (5.9%)
Total	649 (91.6%)	59 (8.3%)	708 (99.9%)

Tele-HHUSL tele-mentored handheld ultrasound.

**Table 5:** Tele-HHUS system compared with other existing systems.

	Real-Time or Asynchronous	Data Transfer Platform	Network	US Type	Portable	Cost	Application Fields
Tele-HHUS system	Real-time	Custom software	Private 4G or 5G network	HHUS	Yes	Low	Wide
Tele-US system	Both	Data collector device	Private network	Conventional US system	No	High	Wide
Telerobotic US system	Real-time	Two subsystems	5G network	Telerobotic US system	No	Very high	Narrow
HHUS system alone	None	None	None	HHUS	Yes	Low	Wide

Tele-HHUS: tele-mentored handheld ultrasound.

## Data Availability

The datasets generated and analyzed during the current study are not publicly available but are available from the corresponding author on reasonable request.

## Abbreviations

Tele-HHUS	Tele-mentored handheld ultrasound;
HHUS	Handheld ultrasound;
US	Ultrasound;
GPs	General practitioners;
WHO	World Health Organization.

## Appendix A

  Full list of participants:
Rural general practitioners:
Fuzhuo Dong (Nanpuxi Town Lianyun Health Center);
Shiliu Zhou (Zhuozhai Village Clinic);
Minyun Shi (Shiyang Branch of Taishun County Hospital of Chinese Medicine);
Shuting Zhang (Shiyang Branch of Taishun County Hospital of Chinese Medicine);
Ningning Lei (Xiaocun Branch of Taishun County People’s Hospital);
Yang Wang (Xiyang Town Health Center);
Dongdong Lei (Sankui Branch of Taishun County People’s Hospital);
Xiaoqian Jun (Sankui Branch of Taishun County People’s Hospital);
Chenmeng Cai (Sankui Branch of Taishun County People’s Hospital);
Qingqing Cai (Sankui Branch of Taishun County People’s Hospital);
Hao Hong (Sankui Branch of Taishun County People’s Hospital);
Chuanji Mei (Sankui Branch of Taishun County People’s Hospital);
Yingxue Shu (Sankui Branch of Taishun County People’s Hospital);
Chunyan Weng (Sankui Branch of Taishun County People’s Hospital);
Xuejing Ye (Sankui Branch of Taishun County People’s Hospital);
Shaobang Li (Baoyang Township Health Center);
Zhangning Wu (Nanpuxi Town Health Center);
Guangwu Wu (Nanpuxi Town Health Center);
Deping Wang (Sixi Branch of Taishun County People’s Hospital);
Xiuxiu Zeng (Pengxi Town Central Health Center);
Limei Li (Pengxi Town Central Health Center);
Hongzu Qiu (Pengxi Town Central Health Center);
Jie Wang (Sixi Town Hengkeng Health Center);
Saie Lei (Siqian Town Central Health Center);
Li Lin (Siqian Town Central Health Center);
Shiyong Luo (Siqian Town Central Health Center);
Yi Wu (Siqian Town Central Health Center);
YaTing Ji (Fengyang Branch of Taishun County People’s Hospital)
Remote experts:
Chengzhong Peng (Shanghai Tenth People’s Hospital);
Huihui Chai (Shanghai Tenth People’s Hospital);
Ruizhong Ye (Zhejiang Provincial People’s Hospital);
Weinan Chen (Zhejiang Provincial People’s Hospital);
Jianchun Li (Zhejiang Provincial People’s Hospital);
Ceng Wang (Zhejiang Provincial People’s Hospital);
Yanming Zhang (Zhejiang Provincial People’s Hospital);
Lizhen Pan (Zhejiang Provincial People’s Hospital);
Zezhou Song (Zhejiang Provincial People’s Hospital).
